# P-1410. Oral Antimicrobial Therapy Offers in Hospitalized Persons Who Inject Drugs who Elect for Self-directed Discharge

**DOI:** 10.1093/ofid/ofae631.1585

**Published:** 2025-01-29

**Authors:** Christen J Arena, Bryce Vanhorn, Rachel M Kenney, Dana M Parke, Geehan Suleyman, Susan L Davis, Michael Veve

**Affiliations:** Eugene Applebaum College of Pharmacy and Health Sciences, Wayne State University and Henry Ford Health, Royal Oak, Michigan; Eugene Applebaum College of Pharmacy and Health Sciences, Wayne State University, Detroit, Michigan; Henry Ford Hospital, Detroit, Michigan; Henry Ford Health, Detroit, Michigan; Henry Ford Health, Detroit, Michigan; Wayne State University, Detroit, Michigan; Henry Ford Health, Detroit, Michigan

## Abstract

**Background:**

Hospitalized Persons Who Inject Drugs (PWID) who elect for self-directed discharge (SDD) are at an increased risk for poor infection outcomes, but there is limited guidance for infection management in this population. National PWID management guidelines suggest considering oral antimicrobial therapy offers (OATO) as soon as patients are clinically stable to avoid lack of antibiotic therapy at discharge. The study purpose is to evaluate infection management in PWID who elect for SDD and to identify characteristics associated with OATO.

Variables Associated with Oral Antimicrobial Offers at Self-directed Discharge

**Figure:**
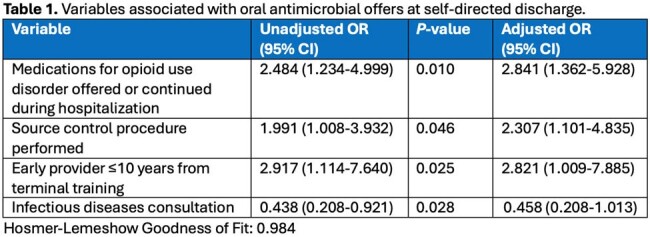

**Methods:**

Retrospective cohort of hospitalized adult PWID with an injection drug use (IDU)-related infection who elected for SDD between 1/1/14-1/31/24 at Henry Ford Health in Michigan. Patients were excluded if they were hospitalized for < 24-hours or if antimicrobial treatment was completed prior to SDD. The primary outcome was the proportion of patients with OATO at or prior to SDD. Secondary outcomes at 30-days included retreatment, infection-related readmission, and all-cause mortality.

**Results:**

150 patients were included; 55 (37%) were OATO patients, 95 (63%) did not receive an offer. Most patients were white (118, 79%), had prior SDD (90, 60%), and were a median (IQR) age of 34 (30-44) years. Skin infections were most common (81, 54%). Patients that received a source control procedure (27 (49%) vs. 31 (33%), *P*=0.05) or care from a provider ≤10 years from terminal training (49 [89%] vs. 70 [74%], *P*=0.04) were more likely to receive an OATO. Patient outcomes were not different between the OATO and no offer groups: infection retreatment 19 (34%) vs. 32 (34%); infection-related readmission 14 (25%) vs. 31 (33%); and all-cause mortality 1 (2%) vs. 3 (3%). Characteristics associated with OATO were prescribing or continuing medications for opioid use disorder during hospitalization, infection source control, and care from providers ≤10 years post-terminal training; infectious diseases consultation had an opposite association (**Table 1**).

**Conclusion:**

Most hospitalized PWID with IDU-related infections with SDD did not receive an OATO. Early career providers more commonly offered oral antimicrobials in PWID with less complicated infection types. Standardizing OATO in PWID at risk for SDD should be considered as a future direction to improve health outcomes.

**Disclosures:**

**Rachel M. Kenney, PharmD, BCIDP**, Medtronic Inc: Spouse is an employee, stockholder **Dana M. Parke, MA**, Pfizer Global Medical Grants & Global Bridges at Mayo Clinic (grant # 70973735): Grant/Research Support|The Kresge Foundation: Grant/Research Support

